# Current situation and effectiveness of palliative care training for staff in an emergency care medical consortium hospital: a cross-sectional study

**DOI:** 10.3389/fmed.2025.1480273

**Published:** 2025-03-31

**Authors:** Liang Zong, Hui Jiang, Huadong Zhu, Jihai Liu, Jun Xu, Xiaohong Ning, Fan Li, Jian Gao, Bo Li, Di Shi, Xin Rao

**Affiliations:** ^1^The Emergency Department, The State Key Laboratory for Complex, Severe and Rare Diseases, Peking Union Medical College Hospital, Chinese Academy of Medical Science and Peking Union Medical College, Beijing, China; ^2^The Department of Geriatrics, Peking Union Medical College Hospital, Peking Union Medical College, Chinese Academy of Medical Sciences, Beijing, China; ^3^The Department of Critical Care Medicine, Zhongnan Hospital of Wuhan University, Wuhan, Hubei, China

**Keywords:** palliative care, cross-sectional study, current situation, emergency department, medical education

## Abstract

**Background:**

The emergency department (ED), usually deemed not the most frequent setting for palliative care (PC), has increasingly been mentioned for its potential critical role in end-of-life patient care. However, how the training affects PC performance remains to be investigated. This study aims to investigate the current PC standard of care and effectiveness of PC training in a Chinese emergency care medical consortium hospital.

**Methods:**

We conducted an anonymous online census targeting the emergency care providers in the consortium hospital. The questionnaire included respondents’ demographics, PC knowledge, PC practice, and whether they have received any PC training. Outcome variables included: confidence in clinical implementation, perceptions about death, and attitudes toward PC implementation with Likert five score rating. Factors associated with better PC knowledge and performance were identified by analysis of the association between rating scores and participant characteristics.

**Results:**

923 staff participated in the study, while 429 (46.5%) received PC training. Training participation was significantly associated with age, education, occupation, rank, working years, and experience of family members’ death (*p* < 0.05). Training improved the total score of knowledge and practice of PC (median 90 vs. 100, *p* < 0.001), the confidence in clinical PC management (confidence score: 36 vs. 40, *p* < 0.001), and attitudes toward PC implementation (attitude score: 37 vs. 40, *p* = 0.048). Offline lecture-based learning was the primary training form in this hospital. The ORs of case-based learning, online lecture video, and community training project to higher total scores were 1.94 (95% CI 1.18–3.17, *p* = 0.009), 2.09 (1.23–3.56, *p* = 0.006) and 0.17 (0.04–0.63, *p* = 0.008), respectively. Meanwhile, cased-based learning, online lecture video, and community training project contributed significantly to the confidence score. So did the lecture offline to the score of perception about death (perception score). The OR of meeting online to attitude score was 1.69 (1.05–2.73, *p* = 0.030).

**Conclusion:**

Palliative care training is associated with better self-rating of PC among ED care providers. However, there is a significant gap for improvement, particularly for the community training programs.

## Introduction

With the accelerated population aging around the globe, a significant proportion of older people are at the end stage of various diseases. Patients with end-stage cancer often have to deal with poor prognoses and live a low quality of life despite incurring significant economic burdens (1). Palliative care (PC) is active, comprehensive medical and psychological care for patients with the life-limiting disease who respond poorly to treatment, including symptom management, providing social, psychological, and spiritual care for patients and families to improve the quality of life before death (2). An increasing number of hospitals in the United States offer PC for end-of-life patients (3). Studies have shown that this medical care not only impacts patient survival (4, 5) but also improves the quality of life (4–6), with evident benefits for patients, families, and society.

In China, the concept of PC was only recently introduced but is gaining traction in the medical community quickly. Despite the heightened awareness, current professional education in medical schools tends to focus on treating somatic diseases but pays less attention to PC training (7), resulting in a lack of communication skills with end-of-life patients and their families, and weak clinical practice of PC (8). In recent years, the Chinese medical community has begun to shift the focus to PC education by introducing various forms of training and discussing the topic in academic journals (9).

Emergency departments (EDs) are one of the most frequently visited medical facilities for patients with life-limiting diseases (10). Unfortunately, many healthcare providers in the ED lack PC knowledge and clinical practices competency (11, 12). Proper palliative care and goal setting for end-of-life patients were often overlooked while clinicians focused on relieving them of their immediate pain and symptoms (11). Studies have shown that PC training in EDs could reduce medical costs and improve quality of life (13, 14). The prerequisite for providing professional PC is high-quality training among medical and non-medical staff (15). However, few studies have examined the current landscape and investigated the effectiveness of PC training in Chinese EDs.

Being overcrowded has challenged EDs in tertiary hospitals in China (16, 17). Attempting to relieve the burden of clinical care, the ED of Peking Union Medical College Hospital (PUMCH) collaborated with a geriatric hospital, Beijing Longfu Hospital (BLH), in 2019 to establish an emergency care hospital consortium. PUMCH ED and BLH have a tight relationship. PUMCH-ED relieves the patients’ urgent situation, and is responsible for candidate patients screening, initial PC treatment and making PC strategy. Then the PC-required patients will be transferred and admitted by BLH, where they receive interdisciplinary comprehensive PC treatments. The consortium would facilitate the referral of end-of-life patients visiting the PUMCH ED to the consortium hospital (BLH), where PC has been implemented in BLH since 2011 and provider PC education started in 2014. We conducted a cross-sectional study on the PC training of the emergency care hospital consortium, aiming to understand training participation, the factors that influence training participation, training effectiveness, and comparing the impact of different training methods on staff’s behaviors.

## Methods

### Study design

We performed a survey using an anonymous online questionnaire. The questionnaire was designed based on a previous study (18). A panel of PC, emergency medicine, and geriatrics experts reviewed and approved the questions, response options, and preferred answers. The questionnaire consisted of respondents’ demographic questions, knowledge and practice of PC, and training requirements for PC. The demographic questions included the respondents’ demographic characteristics, experience with the death of family members, and PC training experience and forms. The knowledge and practice of PC consisted of 3 domains: confidence in clinical implementation, perceptions about death, and attitudes toward PC implementation, in which questions were designed based on the five-points Likert rating scale. The typical Likert scale was developed in 1932 by Rensis Likert to measure attitude (19). Now, it is frequently used in medical education and medical education research (20). Our scale ranged from one point for “not confident at all” or “strongly disagree” to five points for “very confident” or “strongly agree.” The last session, the training requirements for PC, surveyed the demand for further training, weaknesses that call for further improvement, and desired training approaches (Supplementary Material 1). This study was approved by BLH Ethics Committee.

### Study setting and population

This study was conducted at BLH. It was a census which included all staff. A total of 1,094 hospital care providers, including physicians, nurses, technicians, administrators, and other non-clinical staff, were enrolled in the study in May 2021. The study was a cross-sectional survey and did not need sampling, which did not need to calculate the sample size.

### Survey administration

The questionnaire was administered online through the Jinshuju system,^1^ a professional investigation website. The questionnaire’s content was edited online by a trained emergency fellow and checked by another two clinicians to ensure the consistency of the online questionnaire with the actual questionnaire. Questions were pilot tested among clinicians and nurses. The pilot test aimed to find easily misunderstood descriptions and logical errors. We selected 15 physicians and 32 nurses from the emergency department of a tertiary care hospital for the pilot test. After the test, they gave feedback on two poorly described questions, which were rewritten to ensure that similar problems were avoided in the formal study. No logical errors were found in the pilot test. None of the participants in the pilot survey was enrolled in the final study. The questionnaire was posted online by a program administrator of the hospital. Participants submitted this questionnaire anonymously. The collected data were verified electronically and manually for the validity and completeness of all information and answers.

## Data processing and analysis

### Reliability and validity testing of the questionnaire

The reliability and validity assessment were done using IBM SPSS Statistics 25. The reliability of the questionnaire was analyzed using Cronbach’s coefficient alpha method, and the validity was analyzed using exploratory factor analysis (EFA). Kaiser-Meyer-Olkin (KMO) test and Bartlett’s sphericity test were evaluated. KMO > 0.8 and the *p*-value of Bartlett’s sphericity test <0.05 are considered to be suitable for EFA. To identify the number of factors, we employed the eigenvalue>1 as the cut-off value. We used the Varimax as the rotation method. We accepted 0.40 level as a factor loading threshold to consider that a factor is stable.

### Data coding and transformation

The collected data were coded for subsequent statistical analysis. In part II, knowledge and practice of PC, each question was assigned a score of 1–5 on a scale from disagreeing entirely to agreeing completely. Except for indicated questions (see Supplementary Material 1), the preferred answers to most questions were higher scores. For those indicated questions, the scores were coded by the criteria of “6-raw answer” to transform to the range of 1–5. Then, the score of each question was summed up for the total score.

### Data description and hypothesis testing

Continuous or numerical data were described by the median and interquartile range (IQR), and categorical data were described using number and composition ratios. The Mann–Whitney test was used to compare continuous data, and the chi-square test was used to compare categorical data. A two-sided *p* value <0.05 was used as the criterion for a significant difference. The above statistical analyses were performed using IBM SPSS Statistics 25.

### Binary logistic regression

Binary logistic regression was used to analyze the OR of the effect of training forms on the total score. The goodness of fit was evaluated by the Hosmer-Lemeshow test. Scores higher than the median score of all respondents were classified as positive outcome. We treated dummy variables with participation or non-participation in various training forms as dichotomous variables, with participation coded as “1” and non-participation coded as “0.” All variables are kept in the equation.

## Results

### Reliability and validity of the questionnaire

This questionnaire was test filled on May 9, 2021, officially released on May 10, and the last questionnaire was collected on May 18. The KMO value was 0.972, and *p* < 0.001 of Bartlett’s sphericity test, which showed that the data has no inadequacy to carry out EFA. Three factors were meaningful, and the 3 factors explained 77.97% of the variance cumulatively. While factor loadings were between 0.80 and 0.88 for Factor 1, the loadings were 0.54–0.92, 0.64–0.84 for Factor 2 and 3, respectively. The factors’ attribution was aligned with the dimensions we designed. The data showed good structural validity.

### Demographic characteristics

A total of 923 out of 1,094 questionnaires were collected, with an 84.4% response rate. Most respondents were female, accounting for 79.5%, while 3.8% did not disclose their gender. In terms of age, most of the respondents were 18–60 years old, with more than half of them concentrated in the age group of 31–50. The respondents’ highest education was mainly university (40.4%), followed by postgraduate and above (13.0%). Physicians accounted for 26.9% of the respondents, clinical nurses accounted for 36.5%, and the rest were technicians, administrators, and non-clinical staff. Most of the respondents had worked for 20 years or less (76.5%), 79.8% of the respondents had experienced the death of families, and only 7.5% had religious beliefs (Table 1).

**Table 1:** Respondents’ demographic information and its association with palliative care training participation.

	Demographic information (*n* = 923)	Training participation		*P*
		No (*n* = 494)	Yes (*n* = 429)	
Gender				0.362
Male	154 (16.7%)	90 (58.4%)	64 (41.6%)	
Female	734 (79.5%)	387 (52.7%)	347 (47.3%)	
Unknown	35 (3.8%)	17 (48.6%)	18 (51.4%)	
Age				<0.001*
<25	137 (14.8%)	93 (67.9%)	44 (32.1%)	
26–30	157 (17.0%)	83 (52.9%)	74 (47.1%)	
31–40	238 (25.8%)	146 (61.3%)	92 (38.7%)	
41–50	235 (25.5%)	107 (45.5%)	128 (54.5%)	
> = 51	156 (16.9%)	65 (41.7%)	91 (58.3%)	
Education				0.001*
Middle school	108 (11.7%)	37 (34.3%)	71 (65.7%)	
High school	67 (7.3%)	34 (50.7%)	33 (49.3%)	
College	255 (27.6%)	149 (58.4%)	106 (41.6%)	
University	373 (40.4%)	206 (55.2%)	167 (44.8%)	
Master & Doctor	120 (13.0%)	68 (56.7%)	52 (43.3%)	
Occupation				0.021*
Physician	248 (26.9%)	135 (54.4%)	113 (45.6%)	
Nurse	337 (36.5%)	189 (56.1%)	148 (43.9%)	
Administration	73 (7.9%)	46 (63.0%)	27 (37.0%)	
Technician	152 (16.5%)	64 (42.1%)	88 (57.9%)	
Others	113 (12.2%)	60 (53.1%)	53 (46.9%)	
Rank				0.007*
None	42 (4.6%)	28 (66.7%)	14 (33.3%)	
Junior	484 (52.4%)	280 (57.9%)	204 (42.1%)	
Middle	321 (34.8%)	150 (46.7%)	171 (53.3%)	
Vice-senior	57 (6.2%)	26 (45.6%)	31 (54.4%)	
Senior	19 (2.1%)	10 (52.6%)	9 (47.4%)	
Working years				0.007*
<1	54 (5.9%)	40 (74.1%)	14 (25.9%)	
1–5	219 (23.7%)	122 (55.7%)	97 (44.3%)	
6–10	213 (23.1%)	107 (50.2%)	106 (49.8%)	
11–15	141 (15.3%)	77 (54.6%)	64 (45.4%)	
16–20	79 (8.6%)	47 (59.5%)	32 (40.5%)	
> = 20	217 (23.5%)	101 (46.5%)	116 (53.5%)	
Experiences of family members’ death				0.027*
Not have	186 (20.2%)	113 (60.8%)	73 (39.2%)	
Have	737 (79.8%)	381 (51.7%)	356 (48.3%)	
Religion belief				0.462
Not have	854 (92.5%)	460 (53.9%)	394 (46.1%)	
Have	69 (7.5%)	34 (49.3%)	35 (50.7%)	

Data were presented as numbers (%). Statistical analysis was based on the Pearson chi-square test. *Represent significant differences (*p* < 0.05).

### The factors that impact training participation

Among all the respondents, 429 (46.5%) had received PC training. There was a significant difference in training participation rates across age, education, occupation, rank, working years, and experiences of family members’ death subgroups (*p* < 0.05) (Table 1). Older participants are more likely to receive PC training, with the highest participation rate for those 41 years old and above reaching more than 54%. The group with middle school education showed the highest participation rate, while the rest of the education groups all had lower than 50% training participation experiences. The training participation rates of physicians and nurses were 45.6 and 43.9%, respectively, while the highest rate was found in the technician population, reaching 57.9%. The training participation rate of those with junior rank was lower than that of the middle, vice-senior, and senior rank population; training participation was higher among those with experience of family members’ death than those without that (48.3% vs. 39.2%).

### The influence of training experience on knowledge and practice of PC

To evaluate the effectiveness of training, we compared the scores of PC knowledge and practice between participants with or without training. The median score of all respondents was 93 (IQR 72–113). The median score of the trained group was 100 (70–119), while the untrained group was 90 (72–107), which showed significant differences (*p* < 0.001). The overall score for confidence in the clinical implementation of PC subjects (confidence score) was 36 (IQR: 24–48). Respondents’ scores with previous training experience were higher than those without training (40 vs. 36, *p* < 0.001). There was no statistically significant difference between the trained and untrained scores for the view of death subject (view score) (*p* = 0.173). On the attitudes toward PC implementation subject (attitude score), the trained population scored 40 (24–51), a slight increase compared to the untrained population (*p* = 0.048) (Table 2).

**Table 2:** Comparisons differences of scores between respondents with or without training.

	All respondents	Training		*P*
		No	Yes	
Total score	93 (72–113)	90 (72–107)	100 (70–119)	<0.001*
Confidence in clinical implementation of palliative care (confidence score)	36 (24–48)	36 (24–45)	40 (24–54)	<0.001*
Views about death (view score)	16 (12–18)	16 (12–18)	15 (12–18)	0.173
Attitudes toward palliative care implementation (attitude score)	38 (29–49)	37 (31–48)	40 (24–51)	0.048*

Data were presented as median (interquartile range). Statistical analysis was based on Mann–Whitney U test. *Represent significant differences of the scores between respondents with or without training (*p* < 0.05).

Pain control is an essential component and one of the learning objectives of PC. We analyzed the answers to specific questions about pain management (Table 3). Among the six questions, the answer distribution of “Pain management for the end-of-life patients should be given regularly” (Q2) in Table 3 did not show a significant association with training. More than half of respondents believed that “pain at the end-of-life patients is inevitable” (Q1), and most of them had not received specific pain management training. The other three questions were about morphine addiction and symptom control. In the respondents holding perspectives fitting with PC theories, the ratios of whom received training were only 52.2% (Q3), 51.1% (Q5), and 51.1% (Q6).

**Table 3:** Impact of training on perceptions of pain management for the end-of-life patients in palliative care practice.

	Training		*P*
	No (*n* = 494)	Yes (*n* = 429)	
Q1: Pain at the end-of-life patients is not inevitable			
Disagree	332 (56.9%)	251 (43.1%)	
Agree	162 (49.1%)	168 (50.9%)	
Q2: Pain management for the end-of-life patients should be given regularly			0.098
Disagree	363 (55.3%)	294 (44.7%)	
Agree	131 (49.2%)	135 (50.8%)	
Q3: Morphine addiction is not a very serious side effect in end-of-life patients because of the limited duration of survival			0.006*
Disagree	326 (57.1%)	245 (42.9%)	
Agree	168 (47.7%)	184 (52.3%)	
Q4: Pain assessment by patients themselves is more rational and effective than health care providers			0.042*
isagree	340 (55.9%)	268 (44.1%)	
Agree	154 (48.9%)	161 (51.1%)	
Q5: Pain relief should be adequately managed, even if the pain is not caused by end-of-life condition (e.g., tumor)			0.010*
Disagree	296 (57.3%)	221 (42.7%)	
Agree	198 (48.8%)	208 (51.2%)	
Q6: Patients need good control of pain symptoms			
Disagree	289 (57.3%)	215 (42.7%)	0.011*
Agree	205 (48.9%)	214 (51.1%)	

Data were presented as numbers (%). Statistical analysis was based on Pearson chi-square test. *Represent significant differences (*p* < 0.05).

### Training forms and impacts on improving knowledge and practice of PC

**Figure 1:**
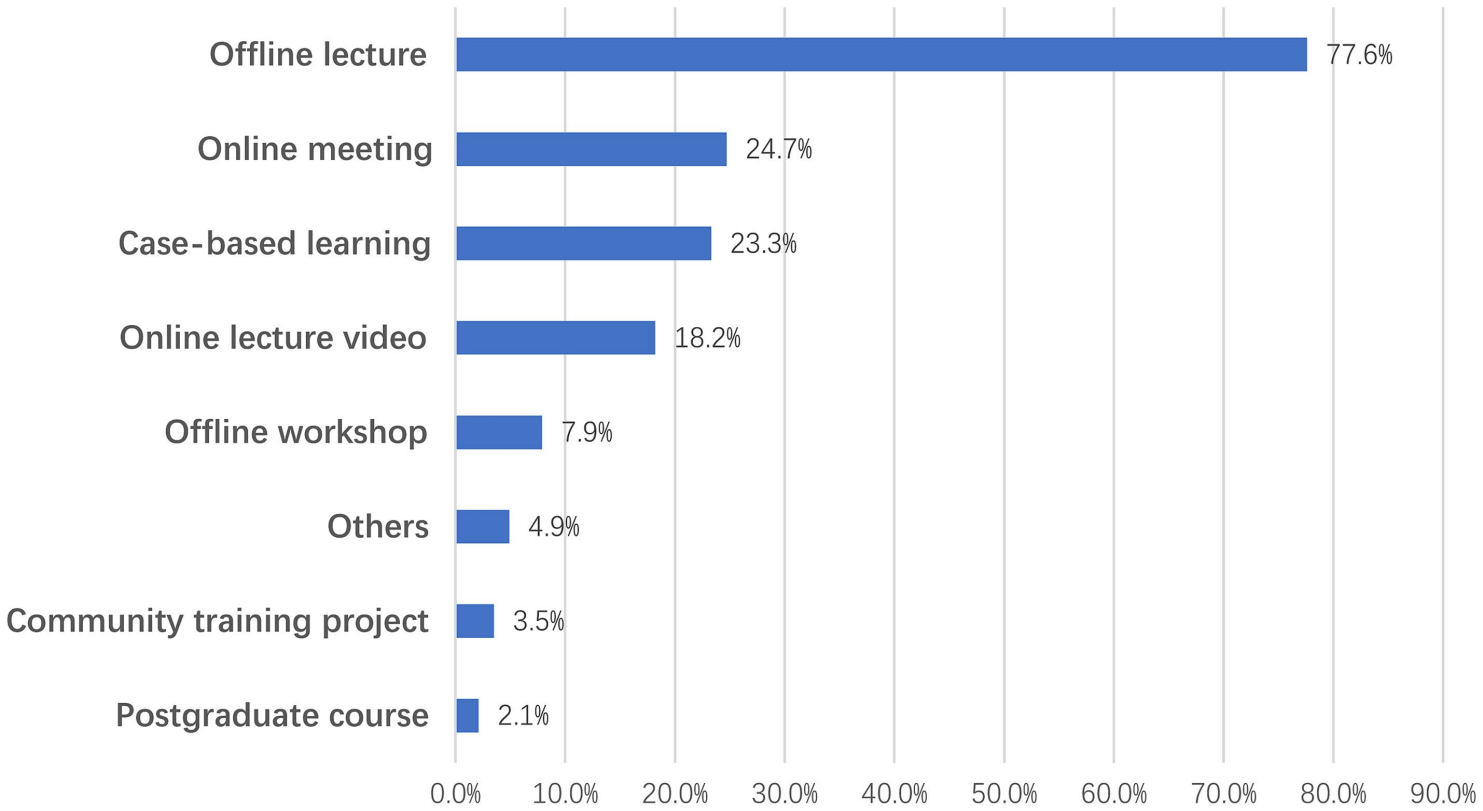
Training forms in which respondents had participated and the ratio (*n* = 429).

The most frequent form of training was offline lectures, with 77.6% of the trained respondents having attended this form of training, followed by online meeting (24.7%), case-based learning (23.3%), online lectures video (18.2%), offline workshops (7.9%), community training projects (3.5%), and postgraduate courses (postgraduate medical courses for preclinical training) (2.1%), and 4.9% of the trainees had participated in other forms of training (Figure 1).

To investigate what training forms were effective, we performed a binary logistic regression analysis as described in “Data process and analysis” part. Scores higher than the median score of all respondents were regarded as positive outcomes. Before logistic analysis, we performed multicollinearity analysis on the dependent variables and their respective variables, and calculated tolerance and variance inflation factors (VIF), and found that tolerance was greater than 0.1 and VIF was less than 10, which could be considered as no significant multicollinearity between their respective variables. Then, the Hosmer-Lemeshow test was performed on the equation, suggesting a good fit of the regression equation (*p* > 0.05).

**Figure 2:**
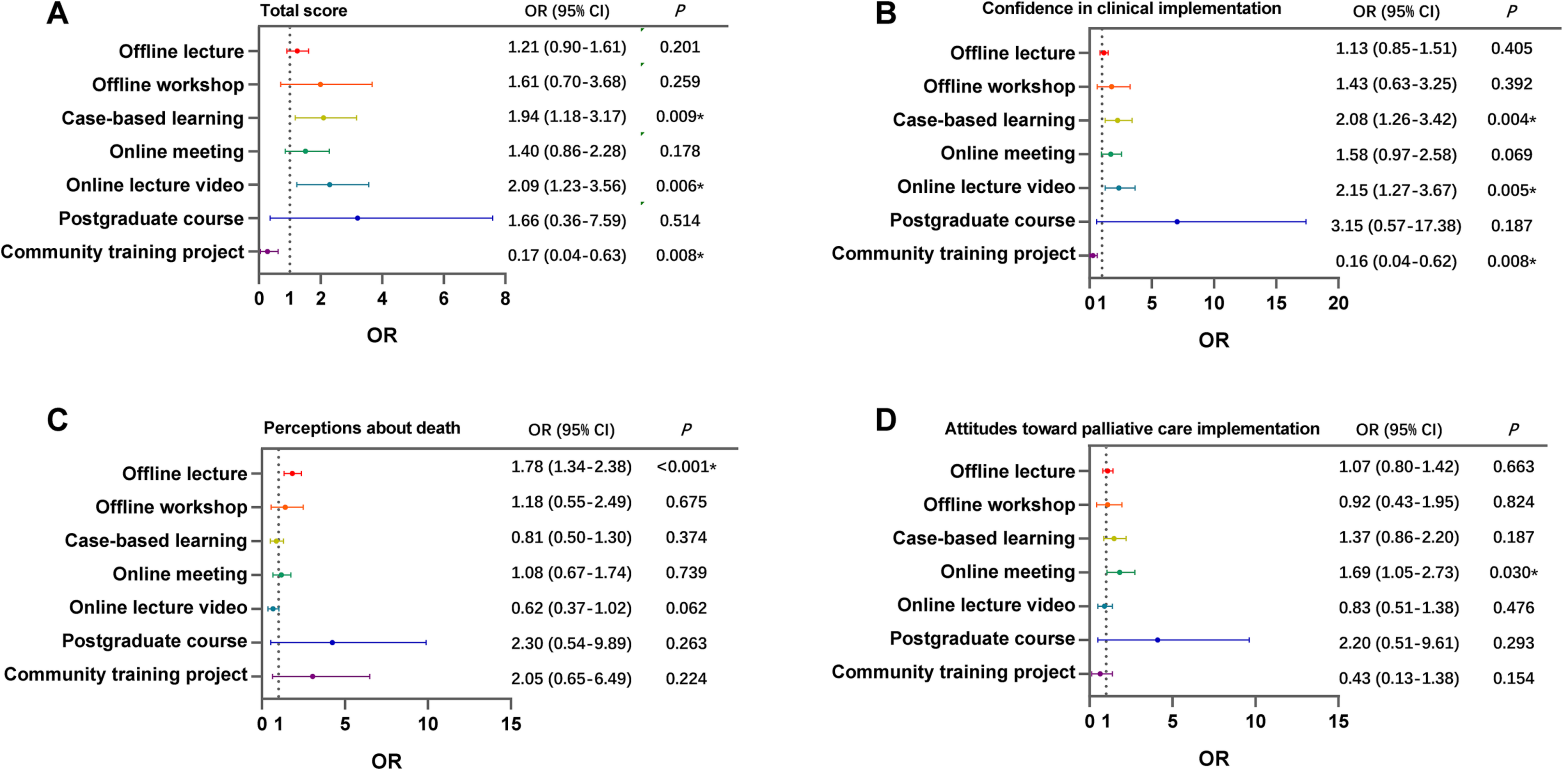
The effect of different training forms on scores of knowledges and practice of palliative care. The figure showed the odds ratio and significance of each training form to the total score **(A)**, the score of confidence in clinical implementation **(B)**, perceptions about death **(C)**, and attitudes toward PC implementation **(D)**. *p* value <0.05 was used as the criterion for a significant difference.

For the total score, the study revealed that the odds ratios (ORs) of receiving a case-based learning, online lecture video, or community training project were 1.94 (95% CI 1.18–3.17, *p* = 0.009), 2.09 (1.23–3.56, *p* = 0.006), and 0.17 (0.04–0.63, *p* = 0.008), respectively (Figure 2A). Then, we analyzed how different training forms contributed to the three subjects. Similar to the total score, the former three training forms significantly influenced confidence in clinical implementation (Figure 2B). The OR of offline lecture to perceptions about death was 1.78 (1.34–2.38, *p* < 0.001) (Figure 2C), indicating that it was able to help participants establish recognition of death with the palliative care principle. Similarly, for the attitudes toward PC implementation subject, online meeting had a statistically significant effect on the total score with an OR of 1.69 (1.05–2.73, *p* = 0.030) (Figure 2D), which had a positive effect on the attitude score.

### Survey on willingness for further training

**Figure 3:**
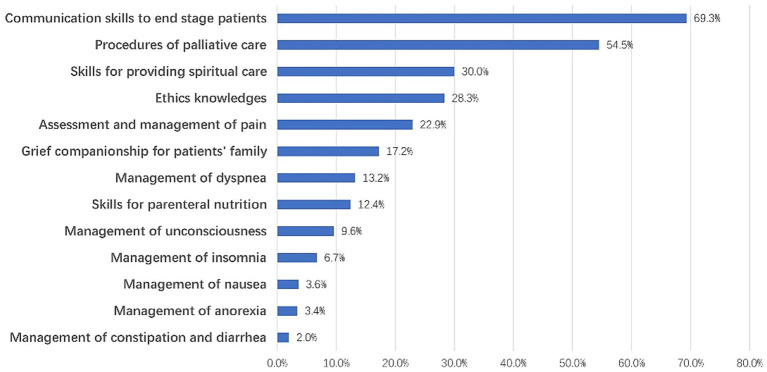
Palliative care skills that respondents felt needed to be upgraded.

We surveyed the respondents’ willingness to participate in training for PC practice, and 773 respondents agreed that emergency care medical consortium hospital staff should be trained for PC, accounting for 83.7% of the total respondents. The top three mentioned areas for improvement were communication skills (69.3%), procedures of PC (54.5%), and skills for providing spiritual care (30.0%) (Figure 3).

## Discussion

PC provides physical and psychological care to patients and families with end-stage and life-limited diseases to improve their quality of life. However, PC is rarely performed in the ED, which may result from a lack of knowledge and training (21, 22). In the PUMCH ED, we have established a referral pathway to transport life-limiting patients suitable for PC to BLH, a hospital that can perform PC. In this study, we conducted a census in BLH to understand the staff’s knowledge, practice, and training implementation in PC and analyze the prevalence of PC education in this hospital and whether previous training experience could enhance clinical PC management.

World Health Organization pointed out that one of the components of a comprehensive approach to PC service was the team working (23). The providers of PC are not only clinicians and nurses but also require a team work (24, 25). Therefore, we considered all hospital potential PC providers and recruited all care providers working in BLH beyond clinicians and nurses.

At BLH, PC training has been taken under diverse methods and facilitated irregularly for the broader workforce beyond clinicians and nurses. All staff was encouraged to participate voluntarily in training. Achieving career goals and related experiences are important motivations for participating in training (26), so clinical needs are associated with a higher willingness to train. Clinical doctors have more frequent contact with patients than hospital managers and have a greater need for palliative care. Therefore, clinical doctors have a stronger willingness to be trained, while managers have a weaker willingness. Similarly, the higher participation of the least educated might be related to the eagerness to gain knowledge.

Due to the large number of patients in the emergency department in China (16, 17), residents are under great work pressure, while senior doctors have more spare time and can better participate in training. This might be one of the reasons why the participation rate of those with vice-senior and middle-ranking is relatively high.

Participation in training showed varying degrees of improvement in PC knowledge and practice scores. This study found that faculties with previous training had significantly higher median scores on both confidence and attitude in PC implementation than faculties without training experience, similar to another retrospective study (15) and two randomized controlled studies (27, 28). Although seldom evidence showed higher self-rating scores were related to a good state of practice and patient outcome, the solid confidence, correct view, and proper attitude were the basis of good performance. Therefore, the higher scores they got, the higher probability they performed well in clinical PC. According to our clinical observation, the physicians receiving PC training performed better than those untrained ones. For example, trained physicians tend to pay more attention to pain control, communication with patients’ families, and collaborating with nurses and social workers to form a comprehensive PC team. Thus, the questionnaire might be able to evaluate clinical performance, but further studies are needed to confirm that.

Our study demonstrated no significant differences in the scores of views about death between trained and untrained people. The most likely reason is that traditional Chinese culture avoids discussing death (29). Thus, palliative care training in China seldom includes death, an essential topic in end-of-life care training. This study suggests that training in the knowledge of death for healthcare professionals needs strengthening.

Although training can improve PC practice to some extent, few scholars have examined the impact of different training methods or forms on training effectiveness. This study showed, interestingly, offline lecture-based learning, the training form with the highest participation of respondents, only showed a positive impact on the perceptions about death subject while having minimal impact on the total score or attitudes and implement of PC. Although offline lecture-based learning is convenient to organize, lectures tend to be theoretical and not recommended for skill training compared to other pedagogies while it provide insufficient opportunities for practice according to educational theories (30). This study showed that video-based online learning positively impacted total and confidence scores. However, the confidence evaluation is not an objective parameter. However, the current findings could hardly confirm its objective effectiveness in clinical PC management.

The case-based learning positively affected the total score and confidence score, but only 23.3% of the staff had participated in this training. The learning process of case-based learning aligns with the clinical workflow, so the trainee will have a more profound impression and can apply the knowledge gained in training to clinical work with more confidence. It has been shown that case-based learning significantly increases the confidence of emergency medicine residents in PC practice and is an effective training method (31). However, the premise of case-based learning is that the audience needs to know PC implementation as a fundamental requirement. Moreover, it is essential for the trainers to adapt the case for designed learning objectives and prepare questions for facilitating training on clinical reasoning as well as decision making, those were either challenges for trainees or trainers, contributing to limit conducted case-based learning in faculty education. On the other hand, BLH is not a teaching hospital, lack of a faculty development training program on teaching competence for trainer was one of reasons limit well designed case-based learning and assure quality for education.

Community-based palliative care has a positive effect on reducing length of stay and increasing patient satisfaction (32). Community training project is a palliative care training program for social workers, family doctors, family nurses, nursing home caregivers, etc., which aims to improve the quality of community-based palliative care. But the effectiveness of the community training project is poor. Firstly, the training objectives of the community training project were inconsistent with the needs of our survey respondents. The community training project targeted community doctors, nurses, and volunteers to conduct palliative interventions in the community, aiming to reduce unnecessary hospitalizations and to guide patients in choosing an appropriate death place (33, 34). However, the object of our survey was the staff of a tertiary hospital, whose work object was patients in the hospital, and their main goal was to improve the life treatment of patients through symptom control and psychosocial support. Therefore, the training content of the community training project was not enough to improve the clinical work confidence of these staff. Second, the training format of the community training project may not be suitable for tertiary hospital staff. The increasing workload of Chinese doctors (35) and the resulting job fatigue (36) lead to a low willingness to train. The distance of the training location and the long training time of the community training project further reduced their desire to train. Even if they have to attend a community training project for some reason, the training effect may not be effective. Third, the training quality of community training projects is worrying. PC has not been widely included in China’s medical education, and there is no unified and standardized teaching material (37). Therefore, even in the few hospitals that have carried out PC courses, its standardization cannot be guaranteed, let alone the community training project. These reasons lead to the poor training effect of the community training projects.

Different elements of the same training method could influence the training effectiveness, which introduced heterogeneity. Moreover, the same method is impossible to fit every trainee. Skilled trainers are good at using different methods for different aims. As a non-technique skill training, PC training could be identified as knowledge skill and attitude sections. According to Miller’s pyramid (38), for each section learning should have different pedagogies, it is impossible to improve PC training with only a single method. Other element we should consider for training program design was time issue, increase the time for case-based learning or simulations and minimize the time for community training project. For residents, theoretical online videos, e.g. the Massive Open Online Courses (MOOC), combined with case-based learning under the supervision of experienced attending physicians could be one of the ways to improve PC practice (27, 28). Recent studies showed that simulation might also be an advanced and effective teaching method (39–41), but it has been rarely applied in PC training. In addition, the training program establish should target to all the populations in the emergency care medical consortium hospital and determine the training content with a tendency according to different learning objectives with various professions of the faculty. PC is a systemic service that includes primary and specialty PC physicians. The competency requirements of the two levels are different, with the former requiring general mastery and the latter geared more toward PC professionals (42).

In addition, unreasonable designs of the training courses may also lead to ineffective outcomes. According to Kirkpatrick’s evaluation model (43), it is necessary to answer the question “what behavior do I want from the trainee” during the course design, and then to suggest what knowledge and skills should be taught to the trainee, and further consider what means can be used to improve the reaction of the audience. Unfortunately, this behavior-oriented training approach is rarely seen in Chinese PC training. The training may be more effective if the curriculum developers consider more about the above issues, not only imparting knowledge.

Regarding the effectiveness of training, this study revealed that it is not promising. Communication with end-of-life patients is a crucial component of PC training, yet our survey found that more than 60% of staff still felt that training in this area needed to strengthen. Another training priority is pain management, in this study, participants demonstrated false perceptions on pain management for end-of-life patients. Despite one of the six questions on pain management having no difference in the distribution of correct answers between respondents with and without training, among other five questions, there was only a slight increase in the percentage of correct answers by trainees, with nearly half of the trainees still answering incorrectly. These findings indicated that the effectiveness of previous trainings was lack of assessment, the quality of the training programs were unacceptable, and calls for well-designed further training programs with precise and definite learning objectives, facilitated by appropriate educations methods.

However, there were limitations in this study. Firstly, we only surveyed among single hospital. Although it was a census, it can only reflect the current situation of PC in acute care hospitals to a certain extent. A more extensive survey is yet to be conducted. Secondly, the participation of hospital staff alone is not enough; patients’ families and community social workers are also essential parts of PC. The degree of cooperation of patients’ families and the practice of community workers also have an important impact on the quality of PC (44, 45), especially during the COVID-19 epidemic, when the value of community-based PC becomes more pronounced (46). Therefore, training patients’ families in PC may also be a way to improve the quality of care. Further research is needed to determine how practical training for patients’ families is and what training methods are more appropriate for families. Thirdly, workers from different departments may have various participations and clinical performance, which still need to be investigated in further study. Fourthly, the study did not describe the details of training methods, such as training time. Since palliative care training varies significantly between countries, the heterogeneity of different training methods might have biased the results. Additionally, self-reported data may introduce biases. Participants may overestimate their knowledge or skills due to social desirability bias. We will use a more objective assessment method in further studies.

## Conclusion

In conclusion, there is a great need for comprehensive promotion of PC, and the current level of PC in clinical practice is insufficient to meet patients’ and families’ needs. Training can improve the self-rating of PC to a certain extent, but not all training forms can achieve that. Incredibly, community training project may negatively affect training outcomes. The content and effectiveness of training still need to be optimized.

## Data Availability

The raw data supporting the conclusions of this article will be made available by the authors, without undue reservation.

## Footnotes

^1^Available at: https://www.jinshuju.net.
