# Supplementary material for: Current situation and effectiveness of palliative care training for staff in an emergency care medical consortium hospital: a cross-sectional study
Source: Front Med (Lausanne). 2025 Mar 31;12:1480273. doi: 10.3389/fmed.2025.1480273 (PMC11994615; doi:10.3389/fmed.2025.1480273)
Supplement: Supplementary file 1 [file Table_1.DOCX]

**A Survey on the Current Situation of Palliative Care
in Beijing Longfu Hospital**

**Description:** Palliative care has grown over the decades since its inception to the present day. In the United Kingdom, the United States, Chinese Taiwan, Singapore, and other places have been flourishing, effectively improving the quality of death of life-limited patients. However, there is an uneven operation of the current situation in various provinces and cities in China. In particular, the promotion and implementation in geriatric specialty hospitals is very rare. Therefore, this study aims to obtain the knowledge and practice of palliative care in our hospital through a survey. Thank you very much for participating in the survey, providing your views and opinions, and we hope to receive your support and cooperation. We take data security very seriously and will never disclose your private information. Let's get started now!

**PART I Basic Information**

1. Gender

〇 Male

〇 Female

〇 Do not want to disclose

2. Age

〇 Below 18

〇 18~25

〇 26~30

〇 31~40

〇 41~50

〇 51~60

〇 Above 60

3. Education level

〇 Middle school or below

〇 High school

〇 College

〇 University

〇 Master or above

4. Hometown

[Blank filling]

5. Occupation

〇 Physician

〇 Nurse

〇 Administration

〇 Technician

〇 Others

6. Department

〇 Tiantongyuan Campus

〇 Beiyuan Campus

〇 Department of Critical Care Medicine

〇 Department of Geriatrics

〇 Neurology

〇 Cardiology / Endocrinology

〇 Hematology

〇 Gastroenterology / Nephrology

〇 Respiratory Medicine

〇 Emergency Medicine

〇 Hemodialysis Unit

〇 Department of Rehabilitation Medicine

〇 Dermatology

〇 Stomatology

〇 Otolaryngology

〇 Ophthalmology

〇 Gynecology

〇 Urology Surgery

〇 Orthopedics

〇 Breast Surgery

〇 General Surgery

〇 Operating Room

〇 Anesthesiology

〇 Department of Preventive Medicine

〇 Infectious Diseases

〇 Acupuncture and Moxibustion

〇 Tui-na

〇 Medical Body Examination

〇 Blood Transfusion

〇 Radiology

〇 Ultrasound Room

〇 Pharmacy

〇 Pathology

〇 Dean's Office

〇 Party Committee Office

〇 Medical Affairs Department

〇 Case Room

〇 Department of Nursing

〇 Department of Science Education

〇 Institute of Geriatric Health

〇 Finance Section

〇 Human Resource Section

〇 Audit and Management Section

〇 Medical Equipment Department

〇 Department of Hospital Medicine

〇 General Affairs

〇 Security Section

〇 Disinfection and Supply Room

〇 Nutrition Department

〇 Field Service Center

〇 Dongcheng District Elderly Rehabilitation Care Guidance Center

〇 Information Center

〇 Outpatient Office

〇 Customer Service Center

〇 Medical Insurance Office

〇 Others

7. Rank

〇 Junior

〇 Middle

〇 Vice-senior

〇 Senior

〇 None

8. Working years

〇 < 1 year

〇 1-5 years

〇 6-10 years

〇 11-15 years

〇 16-20 years

〇 > 20 years

9. Do you have religion belief?

〇 Yes

〇 No

10. Do you have experiences of family members’ death?

〇 Yes

〇 No

11. Have you participated in training of palliative care?

〇 Yes

〇 No

12. Which of the following training forms have you been attended? (Multiple choice, answered only by whom select “yes” in Question 11)

〇 Lecture offline

〇 Workshop offline

〇 Small group case-based learning

〇 Meeting online

〇 Lecture video online

〇 Postgraduate course

〇 Community training project

13. What do you think is your level of knowledge in palliative care? (1 being totally ignorant, 10 being beyond job requirements)

| 1 | 2 | 3 | 4 | 5 | 6 | 7 | 8 | 9 | 10 |
| --- | --- | --- | --- | --- | --- | --- | --- | --- | --- |
| 〇 | 〇 | 〇 | 〇 | 〇 | 〇 | 〇 | 〇 | 〇 | 〇 |

**PART II Knowledge and Practice of Palliative Care**

14. How confident are you when communicating with patients and families and implementing clinical care? (On a scale of 1-5, 1 being not confident at all, 5 being confident)

| Questions | 1 | 2 | 3 | 4 | 5 |
| --- | --- | --- | --- | --- | --- |
| Answering questions from patients and families related to the dying process | 〇 | 〇 | 〇 | 〇 | 〇 |
| Grief companionship | 〇 | 〇 | 〇 | 〇 | 〇 |
| Inform patients and families with applying hospice and palliative care related services | 〇 | 〇 | 〇 | 〇 | 〇 |
| Discussion patient's wishes with patients and families | 〇 | 〇 | 〇 | 〇 | 〇 |
| Guidance on the environment options for death | 〇 | 〇 | 〇 | 〇 | 〇 |
| Introducing side effects of medications to patients | 〇 | 〇 | 〇 | 〇 | 〇 |
| Reasonable management of patient's pain symptoms | 〇 | 〇 | 〇 | 〇 | 〇 |
| Reasonable response and management of patients with terminal delirium | 〇 | 〇 | 〇 | 〇 | 〇 |
| Reasonable response and management of patients with respiratory distress | 〇 | 〇 | 〇 | 〇 | 〇 |
| Reasonable response and management of patient's nausea/vomiting symptoms | 〇 | 〇 | 〇 | 〇 | 〇 |
| Reasonable management of patients with difficulty in defecation and constipation | 〇 | 〇 | 〇 | 〇 | 〇 |
| Meeting needs of patients with incurable conditions and limited management | 〇 | 〇 | 〇 | 〇 | 〇 |

15. How do you know about the process of dying? (On a scale of 1 to 5, with 1 being completely disagree and 5 being completely agree)

| Questions | 1 | 2 | 3 | 4 | 5 |
| --- | --- | --- | --- | --- | --- |
| * Health care workers can offer little help to patients and families during this time | 〇 | 〇 | 〇 | 〇 | 〇 |
| * I am uncomfortable for taking care of end-of-life patients | 〇 | 〇 | 〇 | 〇 | 〇 |
| * Caring for an end-of-life patient and talking to the patient's family makes me uncomfortable | 〇 | 〇 | 〇 | 〇 | 〇 |
| * I am uncomfortable with the process of passing away | 〇 | 〇 | 〇 | 〇 | 〇 |
| * End-of-life patients should be given nasal tubes to avoid starvation | 〇 | 〇 | 〇 | 〇 | 〇 |
| Hospital is not the best place to pass away | 〇 | 〇 | 〇 | 〇 | 〇 |

16. Your attitude toward palliative care? (On a scale of 1-5, with 1 being strongly disagree and 5 being strongly agree)

| Questions | 1 | 2 | 3 | 4 | 5 |
| --- | --- | --- | --- | --- | --- |
| * Pain at the end-of-life patients is inevitable | 〇 | 〇 | 〇 | 〇 | 〇 |
| * Pain management for the end-of-life patients should be given on an as-needed | 〇 | 〇 | 〇 | 〇 | 〇 |
| Treatment of the end-of-life patients should include spiritual care | 〇 | 〇 | 〇 | 〇 | 〇 |
| Palliative care should be the standard strategy for the end-of-life patients | 〇 | 〇 | 〇 | 〇 | 〇 |
| Patients have and should have the right to determine their own treatment strategy and the extent of their treatment | 〇 | 〇 | 〇 | 〇 | 〇 |
| Morphine addiction is not a very serious side effect in end-of-life patients because of the limited duration of survival | 〇 | 〇 | 〇 | 〇 | 〇 |
| * Palliative cares should ONLY be discussed openly with the patient and family if no further treatment strategy can be made | 〇 | 〇 | 〇 | 〇 | 〇 |
| * Pain assessment by health care providers is more rational and effective than patients’ self-rating | 〇 | 〇 | 〇 | 〇 | 〇 |
| Pain relief should be adequately managed, even if the pain is not caused by end-of-life condition (e.g., tumor) | 〇 | 〇 | 〇 | 〇 | 〇 |
| Patients have the right to determine the extent of their psychosocial intervention | 〇 | 〇 | 〇 | 〇 | 〇 |
| * The family physician is the best person to make end-of-life care decisions | 〇 | 〇 | 〇 | 〇 | 〇 |
| Patients need good control of pain symptoms | 〇 | 〇 | 〇 | 〇 | 〇 |

**PART III Training Requirements**

17. I believe that geriatric hospital staff should be trained in palliative care. (On a scale of 1 to 5, with 1 being strongly disagree and 5 being strongly agree)

〇 1

〇 2

〇 3

〇 4

〇 5

18. In your opinion, among the following options, the most important element that needs to be upgraded is (Please select no more than 3 options) (Choose 1~3 items)

〇 Communication skills for end-stage patients

〇 Ethics-related knowledges

〇 Procedures of palliative care

〇 Management of unconsciousness

〇 Assessment and management of pain

〇 Management of dyspnea

〇 Management of anorexia

〇 Management of insomnia

〇 Management of nausea

〇 Skills for providing spiritual care

〇 Management of symptoms of constipation and diarrhea

〇 Skills for parenteral nutrition

〇 Grief companionship for the patients’ families

19. Which of the following forms of study would you like to participate in (select 1~3 options)

〇 Special Lectures

〇 Short-term workshops (less than 1 week)

〇 Case discussions

〇 Longer engagement series (more than 1 week)

〇 Visits for further training

〇 Others Description

* The scores of these indicating questions were coded with the criteria “6-raw answer”. The indicators were not shown in the releasing questionnaire.
